# Evaluation of Differences in Temporal Synchrony Between Brain Regions in Individuals With Autism and Typical Development

**DOI:** 10.1001/jamanetworkopen.2018.4777

**Published:** 2018-11-16

**Authors:** Jace B. King, Molly B. D. Prigge, Carolyn K. King, Jubel Morgan, Douglas C. Dean, Abigail Freeman, Joaquin Alfonso M. Villaruz, Karen L. Kane, Erin D. Bigler, Andrew L. Alexander, Nicholas Lange, Brandon A. Zielinski, Janet E. Lainhart, Jeffrey S. Anderson

**Affiliations:** 1 Department of Radiology and Imaging Sciences, University of Utah, Salt Lake City; 2 Interdepartmental Program in Neuroscience, University of Utah, Salt Lake City; 3 Department of Pediatrics, University of Utah, Salt Lake City; 4 Waisman Center, University of Wisconsin–Madison, Madison; 5 Department of Psychiatry, University of Wisconsin–Madison, Madison; 6 Psychology Department and Neuroscience Center, Brigham Young University, Provo, Utah; 7 Department of Medical Physics, University of Wisconsin–Madison, Madison; 8 McLean Hospital and Department of Psychiatry, Harvard University, Cambridge, Massachusetts; 9 Department of Neurology, University of Utah, Salt Lake City; 10 Department of Bioengineering, University of Utah, Salt Lake City

## Abstract

**Importance:**

Despite reports of widespread but heterogeneous atypicality of functional connectivity in individuals with autism, little is known regarding the temporal dynamics of functional brain connections and how they relate to autistic traits.

**Objective:**

To investigate differences in temporal synchrony between brain regions in individuals with autism and those with typical development.

**Design, Setting, and Participants:**

This cohort study, conducted at the University of Utah, included 90 adolescent and adult male participants. A larger sample from the multisite Autism Brain Imaging Data Exchange (ABIDE) was also used as a replication sample. The study includes data acquired between December 2016 and April 2018. Aggregate data included in the replication sample were released to the public in August 2012 (ABIDE I) and June 2016 (ABIDE II). Data analysis were conducted between January 2018 and April 2018.

**Exposures:**

Male individuals diagnosed as having autism (n = 52) and typically developing male individuals (n = 38).

**Main Outcomes and Measures:**

Long duration (30 minutes/individual) of multiband, multiecho functional magnetic resonance imaging was acquired to estimate functional connectivity between brain regions. Sustained connectivity, a measure of functional connectivity duration, as well as lagged temporal dynamics related to functional connectivity, were compared between groups for 361 gray matter regions of interest and a 17-network parcellation. Lagged findings were replicated in the larger ABIDE sample (n = 1402). Sustained connectivity findings were also associated with behavioral and cognitive variables.

**Results:**

In 52 males with autism (mean [SD] age, 27.73 [8.66] years) and 38 control males with typical development (mean [SD] age, 27.09 [7.49] years), increases in both sustained and functional connectivity at several lags were found in individuals with autism compared with the control group. Group differences in functional connectivity were replicated in the larger ABIDE data set at a 6-second lag. Measures of symptom severity in individuals with autism were positively associated with sustained connectivity values. In the control group, sustained connectivity was negatively associated with cognitive processing. A replication sample (n = 1402) composed of 579 individuals with autism (80 female and 499 male; mean [SD] age, 15.08 [6.89] years) and 823 in the control group (211 female and 612 male; mean [SD] age, 15.06 [6.79] years) from the ABIDE data set was also analyzed.

**Conclusions and Relevance:**

Whereas the magnitude of functional connectivity in autism is variable across brain regions, participant samples, and development, prolonged temporal synchrony of functional connections is reproducibly observed in autism, suggesting a potential mechanism for core symptoms.

## Key Points

**Question:**

Do individuals with autism show atypical duration of brain functional connections?

**Findings:**

In this cohort study of 52 individuals with autism and 38 typically developing participants and a replication study of 1402 participants in a brain imaging database, increased durations of functional connections in autism were found in both distributed networks and individual brain regions, which were associated with metrics of disease severity.

**Meaning:**

Persistence of brain connectivity in autism may limit the ability to rapidly shift from one brain state to another and contribute to the pathophysiology of autism.

## Teaser

This cohort study investigates the differences in temporal synchrony between brain regions in individuals with autism and those with typical development.

## Introduction

Autism has been hypothesized to exemplify a disorder of brain connectivity, yet findings related to atypical functional connectivity in autism have been divergent, including reports of both underconnectivity and overconnectivity.^1^ Most studies examining functional connectivity magnetic resonance imaging (MRI) in autism use static functional connectivity, which treats brain activity as stationary over an extended period of time. While there are some replicated findings such as decreased left and right homotopic connectivity,^2,3^ atypical connectivity most prevalent within default and salience network regions,^4,5,6^ increased corticostriatal connectivity,^7,8^ and idiosyncratic differences from typical development of connectivity in autism,^9,10,11^ the spatial distribution and effect size of connectivity abnormalities are characterized more by heterogeneity than by consistent between-group differences. Thus, increasing importance has been placed on exploring the dynamic relationship of functional connectivity in autism.^12,13,14^

A number of methods have been proposed to assess the temporal components of resting-state functional MRI (fMRI) data, or chronnectomics, which incorporate time-varying patterns of connectivity.^13^ As part of a larger analysis exploring the relationship between dynamic functional connectivity patterns and brain development using chronnectomics, the study by Rashid et al^14^ reported longer dwell times related to a globally disconnected state in youths with higher autistic traits, while youths with fewer autistic traits demonstrated increased dwell times in a default network modularized state. Similar aberrant temporal dynamics were reported in individuals with autism in the study by Watanabe and Rees,^15^ who found decreased transitions between network brain states in adults with autism compared with typical development in an energy landscape analysis.

One recently introduced method for probing the temporal dynamics of functional connectivity is sustained connectivity MRI, which measures how long, on average, functional connectivity persists between brain regions or networks, yielding a gauge of how transient or sustained a given connection is maintained.^16^ Sustained connectivity provides insight into separable components of cognition when compared with static functional connectivity. For instance, the study by King and Anderson^16^ showed a significant negative relationship between processing speed and sustained connectivity in a large sample of typically developing adults. This relationship with processing speed was not evident with static traditional functional connectivity. Processing speed is one aspect of cognition that is impaired in individuals with autism compared with typical development.^17,18^ Despite impaired processing speed not being a direct symptom of autism, processing speed abnormalities have been linked with autism-related traits.^18,19^ Taken together, these data suggest that in individuals with autism, sustained connectivity would be increased and that temporal synchrony differences in functional connectivity in individuals with autism would be associated with symptom severity.

We studied multiband, multiecho resting-state fMRI scans from a well-characterized longitudinal adolescent and adult sample of individuals with autism vs typical development to explore the relationship between a diagnosis of autism and temporal differences in brain synchrony. We hypothesized aberrant prolonged synchrony across brain regions and networks in individuals with autism beyond those explained by traditional measures of functional connectivity. Analyses were conducted using a whole-brain approach without a priori specification of which networks or connections to investigate. Furthermore, we hypothesized that these temporal differences in synchrony would be differentially associated with cognition and autistic traits.

## Methods

### Participants

This study was approved by the institutional review board at the University of Utah. Study participants were recruited from the community and received remuneration for their voluntary participation. This study followed the Strengthening the Reporting of Observational Studies in Epidemiology (STROBE) reporting guideline. All study participants provided written informed assent or consent prior to study participation with parental or legal guardian consent required of all study participants younger than 18 years. Participants included in this study were part of a larger and ongoing longitudinal study that began in 2003 aimed at investigating brain development across the adolescent and adult life span in individuals with autism. Methods used to acquire and analyze the resting-state fMRI data reported on in this study had not been used during any previous longitudinal study time points and incorporate data acquired between December 2016 and April 2018 and analyzed between January 2018 and April 2018. Autism diagnoses were established for each participant on initial inclusion into the longitudinal sample, based on the Autism Diagnostic Observation Schedule (ADOS)–Generic^20^ (earlier entries) or the second edition of the ADOS-2^21^ (later entries), Autism Diagnostic Interview–Revised,^22^ the *Diagnostic and Statistical Manual of Mental Disorders*, fourth edition,^23^ the *Diagnostic and Statistical Manual of Mental Disorders*, fifth edition,^24^ and *International Classification of Diseases, Tenth Revision* criteria. For the current time point in the longitudinal sample, the ADOS-2 was administered to all individuals with autism spectrum disorder. Detailed demographics of participants are included in the Table.

**Table.** Cohort Demographics

Variable	Mean (SD) [Range]		*d*	*t*	*P* Value
	Controls (n = 38)	Autism (n = 52)			
Age, y	27.09 (7.49) [16.33 to 46.9]	27.73 (8.66) [15.33 to 57.92]	0.08	−0.36	.72
Head circumference, cm	57.87 (1.39) [55 to 61]	58.14 (2.77) [53 to 65]	0.12	−0.61	.55
VIQ^a^^,^^b^	117.79 (12.57) [84 to 140]	105.37 (20.25) [61 to 142]	0.70	3.38	<.001
PIQ^a^^,^^b^	115.62 (13.79) [77 to 136]	104.06 (19.16) [67 to 150]	0.67	2.85	.01
FSIQ^b^^,^^c^	118.10 (12.75) [78 to 135]	105.45 (19.83) [60 to 150]	0.73	3.51	<.001
Trail Making Test^d^					
A time	20.56 (8.91) [9 to 56]	32.42 (19.09) [13 to 128]	0.75	−3.87	<.001
B time	49.64 (24.77) [19 to 151]	79.41 (54.90) [23 to 310]	0.66	−3.40	.001
B − A	29.08 (21.74) [7 to 122]	46.99 (43.91) [−21 to 182]	0.49	−2.50	.02
SRS-SCI raw^e^	19.53 (16.65) [1 to 70]	67.21 (26.65) [14 to 123]	2.10	−10.42	<.001
SRS-RRB raw^f^	3.87 (4.91) [0 to 23]	15.88 (6.95) [3 to 31]	1.97	−9.61	<.001
SRS-total raw^g^	23.39 (19.41) [2 to 87]	83.10 (32.92) [19 to 150]	2.15	−10.76	<.001
Initial					
ADOS-SA CSS^h^^,^^i^	NA	8.14 (1.43) [5 to 10]	NA	NA	NA
ADOS-RRB CSS^h^^,^^i^	NA	7.50 (1.99) [1 to 10]	NA	NA	NA
ADOS total score CSS^i^	NA	8.17 (1.56) [5 to 10]	NA	NA	NA
Current					
ADOS-SA CSS	NA	7.16 (2.87) [1 to 10]	NA	NA	NA
ADOS-RRB CSS	NA	7.41 (2.16) [1 to 10]	NA	NA	NA
ADOS total score CSS	NA	7.20 (2.91) [1 to 10]	NA	NA	NA

Abbreviations: ADOS, Autism Diagnostic Observation Schedule; CSS, Calibrated Severity Score; FSIQ, full-scale IQ; NA, not available; PIQ, performance IQ; RRB, restricted and repetitive behaviors; SA, social affect; SCI, Social Communication Index; SRS, Social Responsiveness Scale; VIQ, verbal IQ.

^a^ Controls = 29.

^b^ Participants with autism = 51.

^c^ Controls = 31.

^d^ Controls = 33.

^e^ Scores range from 0 to 159 with higher scores indicating higher level of impairment.

^f^ Scores range from 0 to 36 with higher scores indicating higher level of impairment.

^g^ Scores range from 0 to 195 with higher scores indicating higher level of impairment.

^h^ Participants with autism = 50.

^i^ Scores range from 1 to 10 with higher scores indicating higher level of impairment.

### Imaging Data

Study design this current time point fMRI analysis emphasized 3 goals: (1) longer aggregate imaging time for improved accuracy of results for each participant,^25^ (2) high temporal resolution and exclusion of high-motion individuals to isolate head motion artifacts,^26,27^ and (3) use of multiecho technique to facilitate discrimination of nonphysiological artifacts.^28^ Imaging data were acquired at the Utah Center for Advanced Imaging Research using a Siemens Prisma 3-T MRI scanner (80 mT/m gradients) with a Siemens 64-channel head coil. Structural images consisted of a MP2RAGE sequence with isotropic 1-mm resolution (repetition time [TR] = 5000 milliseconds, echo time [TE] = 2.91 milliseconds, and inversion time = 700 milliseconds). Resting-state functional images were acquired using a multiband, multiecho, echo-planar imaging sequence (TR = 1553 milliseconds; flip angle = 65°; inplane acceleration factor = 2; fields of view = 208 mm; 72 axial slices; resolution = 2.0 mm isotropic; multiband acceleration factor = 4; partial Fourier = 6/8; bandwidth = 1850 Hz; 3 echoes with TEs of 12.4 milliseconds, 34.28 milliseconds, and 56.16 milliseconds; and effective TE spacing = 22 milliseconds). Two resting-state acquisitions of 590 volumes (time series duration = 15 minutes, 27 seconds each; 1 left to right and 1 right to left) were acquired along with pulse and respiration waveform data. In advance of resting-state scan acquisition, individuals were instructed to rest with eyes open while letting their thoughts wander.

### Data Analysis

#### Utah Cohort

Only participants with both resting-state runs and available respiratory data waveforms were included in this analysis. Analyses were conducted in the MATLAB computing environment (The MathWorks, Inc). Structural data were processed using FreeSurfer, version 6.0.0 (http://surfer.nmr.mgh.harvard.edu/).

Analysis of resting-state data was completed using a multiecho independent component analysis (ME-ICA) using the Analysis of Functional Neuroimages (http://afni.nimh.nih.gov) ME-ICA package.^29^ Blood oxygen level–dependent (BOLD) percent signal change increases linearly with TE. The use of ME-ICA decomposes resting-state time series data into independent components and either accepts or rejects components based on their similarity in signal amplitude decay in relation to TE. This method acts to separate and remove sources of noise from the signal that do not track with TEs such as motion-related artifacts; see, for instance, the study by Kundu et al.^30^ Following ME-ICA processing, the time series for each voxel was detrended and then detrended respiratory signal was regressed from the time series to mitigate any remaining physiologic artifacts,^28^ including respiration volume per time and respiratory response function,^31^ each sampled at 0 lag and −4.5 to +4.5 seconds lag from the BOLD time courses for a total of 6 regressors. Volumes before and after root mean square head motion greater than 0.2 mm were censored using motion parameters provided by the ME-ICA pipeline by treating these points as missing data.^27^ Preprocessing was completed for 108 study participants. Visual inspection of ME-ICA output for each participant was then conducted by 2 raters (J.B.K. and J.S.A.), resulting in removal of 7 participants. Participants who had less than half of the original 590 volumes (for each scan) remaining following motion censoring were also removed (n = 11), resulting in a total of 90 participants included in the results.

Following completion of preprocessing, resting-state fMRI data were analyzed on both a 17-network level^32^ as well as a finer-grained brain parcellation scheme as previously described.^16^ Briefly, time series data from each of the 17 distributed brain networks were extracted for analysis with each network treated as a single region of interest (ROI).

The finer-grained parcellation consisted of 333 regions in the cerebral cortex,^33^ 14 participant-specific subcortical regions from FreeSurfer derived segmentation^34^ (bilateral thalamus, caudate, putamen, amygdala, hippocampus, pallidum, and nucleus accumbens), and 14 bilateral cerebellar representations of a 7-network parcellation.^35^ When combined, this parcellation scheme incorporates major cortical, subcortical, and cerebellar gray matter ROIs numbering 361 regions in total.^16,36^

General linear models were used to compare functional connectivity between groups controlling for age and mean head motion. Multiple comparison corrections were completed using false discovery rate (FDR). Findings meeting FDR adjusted *P* value (q[FDR]) less than .05 were considered significant. In some instances, uncorrected *P* values (<.05) are provided for informational purposes related to the scope of the findings as they present across the brain.

**Figure 1.**
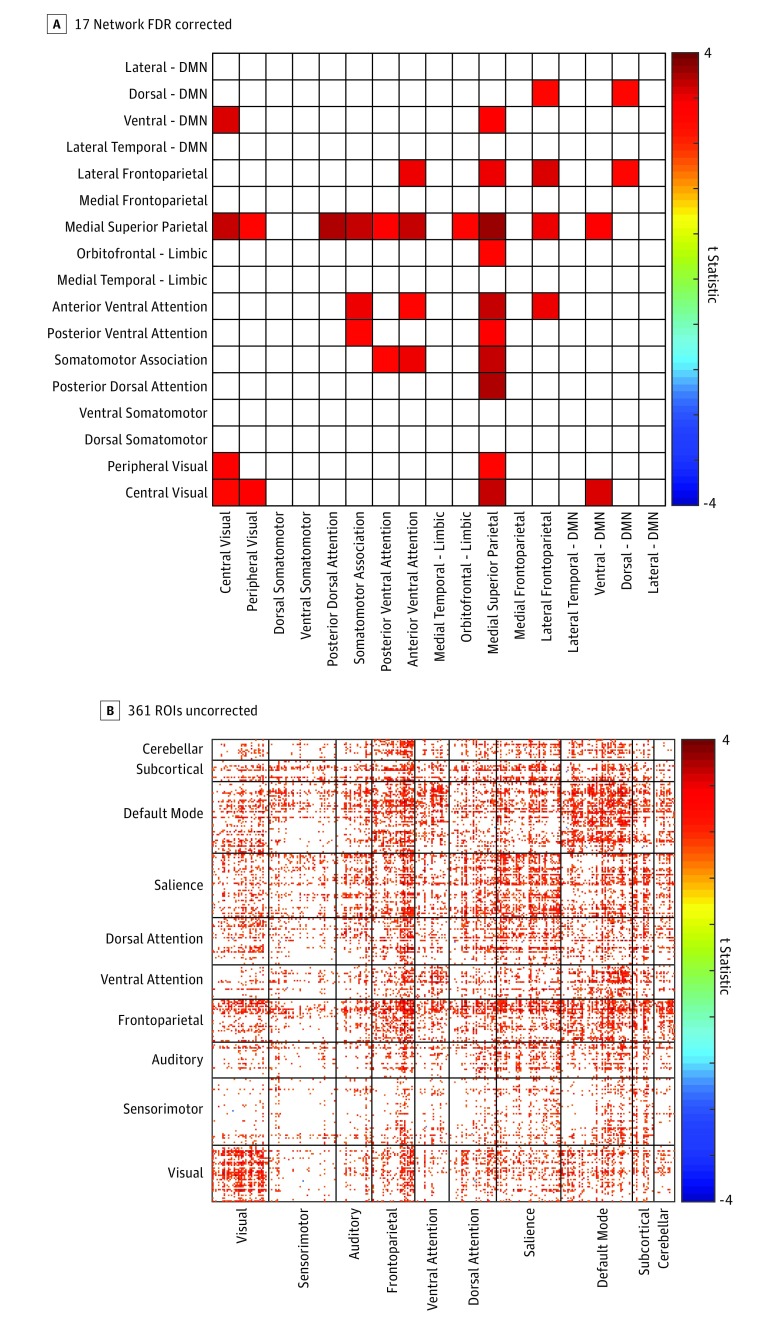
Sustained Connectivity Is Significantly Increased in Individuals With Autism Distribution of increased sustained connectivity values in individuals with autism relative to those in the control group across a 17-network parcellation (false discovery rate [FDR]–adjusted *P* < .05) (A) and across 361 gray matter regions of interest (ROIs) (uncorrected, *P* < .05) (B). DMN indicates Default Mode Network.

To determine the strength of functional connectivity between ROIs across time, 2 temporal methods of analysis were conducted. First, sustained connectivity values were calculated.^16^ For 1 connection involving 2 time series of BOLD measurements, cross-correlation curves were constructed by using the Pearson correlation coefficient between the 2 time series at each of −20 to +20 lags by shifting one or the other of the time points forward in time by 1 volume. For a graphical representation of this process, see the study by King and Anderson^16^ (Figure 1). Data points from volumes before and after volumes with a mean head motion value of 0.2 mm or greater were treated as missing data in sustained connectivity MRI calculations analogous to volume censoring or scrubbing in traditional functional connectivity analysis. Cross-correlation curves were calculated independently for each scan for each individual between pairs of time series for each of the 17 networks and 361 ROIs using the resting-state acquisition repetition time (TR = 1553 milliseconds) as lag values.

The resulting cross-correlation curves were then interpolated using a cubic spline function. Sustained connectivity values were defined as the full width at half maximum value of the cross-correlation curve, with the peak defined as the maximal absolute value of the cross-correlation curve over the interval between −2 to +2 seconds lag (±3.1 seconds).^16^ This allowed the peak to be either positive or negative.

Second, Pearson correlation coefficients were compared between groups at each lag to explore fluctuations across time. Average Pearson correlation values across all pairs of 361 ROIs were computed for each individual at each lag. These values were then compared between groups at 0 lag and at each lag of −20 to +20 lags (±20 TRs).

Analyses were also conducted on the relationship between autism symptom severity with sustained connectivity values across time for the 361 gray matter ROIs and the 17-network parcellation using a linear regression model controlling for age and mean head motion. Findings meeting q[FDR] less than .05 were considered significant. In individuals with autism, symptom severity was assessed using ADOS-2^21^ calibrated severity scores (social affect, restricted repetitive behaviors, and total), based on the revised ADOS Module 4 algorithm,^37^ and the Social Responsiveness Scale (SRS) total raw score, social communication and interaction SRS, and restricted and repetitive behaviors SRS subscale raw scores.^38^ The Trail Making Test (TMT) was administered for both individuals with autism and those in the control group. Total time to completion for the TMT parts A (TMT-A) and B (TMT-B) are investigated as well as the difference in completion times.^39^

### Replication Study

Data from the Autism Brain Imaging Data Exchange (ABIDE) were used to replicate lag-based findings in the current sample. Due to variability in data quality and low temporal resolution (high-repetition time), sustained connectivity values were not calculated in the replication sample as connectivity width has the effect of amplifying outliers in such data. For details on site specific acquisition data within the ABIDE sample (eTable in the Supplement).^40^ Preprocessing of the ABIDE 1 and 2 fMRI BOLD data was performed in Matlab (The MathWorks, Inc) using Statistical Parametric Mapping, version 12 (SPM12; Wellcome Trust Centre for Neuroimaging). Each participant’s BOLD images were realigned and coregistered to their individual magnetization prepared rapid gradient echo anatomic image sequence. Phase-shifted soft-tissue correction^41^ was used to remove regressors obtained from subject motion parameters, degraded white matter, degraded cerebrospinal fluid, and soft tissues of the face and calvarium. Volume censoring (scrubbing) was performed for root-mean-square head motion greater than 0.3 mm.^27^ Individual sites within the ABIDE data set with fewer than 10 participants per group remaining after stringent visual inspection quality assurance were dropped from further analysis. Following quality control measures, a total of 1402 participants remained for further analysis, including 579 individuals with autism and 823 control individuals. Cross-correlation curves were computed using the TR for each individual site, with cubic spline interpolation to identify lagged correlation at 0 lag and positive and negative lags. Since each site potentially used a different TR, we used interpolation of the cross-correlation curves to identify comparable lag values in seconds for each site for pooled group comparisons. Statistical comparisons for the ABIDE data set included age, sex, mean head motion, and a binary variable representing each of the 25 sites used in the analysis as covariates. General linear models were used to compare functional connectivity between groups controlling for age, mean head motion, and site. Each site was treated as a separate binary variable in the general linear model to covary by site. Multiple comparison corrections were completed using FDR. Findings meeting q[FDR] less than .05 were considered significant, calculated over all *P* values for a given test.

## Results

### Demographic and Clinical Results

Sustained connectivity and synchrony of brain regions across time using resting-state data were compared between 52 males with autism (mean [SD] age, 27.73 [8.66] years) and 38 control males with typical development (mean [SD] age, 27.09 [7.49] years). Groups were matched for age, but not full-scale IQ (*t*_80_, −3.170; *P* < .001). Twenty-one participants with autism reported being on a psychoactive medication at the time of scan (stimulant, 8; antidepressant, 10; atypical neuroleptic, 5; mood stabilizer, 2; antianxiety, 2; other psychoactive medication, 5; and multiple medications, 14). Autistic traits and cognitive functioning were also evaluated (Table). A replication sample composed of 579 individuals with autism (80 female and 499 male; mean [SD] age, 15.08 [6.89] years) and 823 in the control group (211 female and 612 male; mean [SD] age, 15.06 [6.79] years) from the ABIDE data set was also analyzed.

### Between-Group Differences in the Sustained Connectivity and Synchrony Across Brain Regions

**Figure 2.**
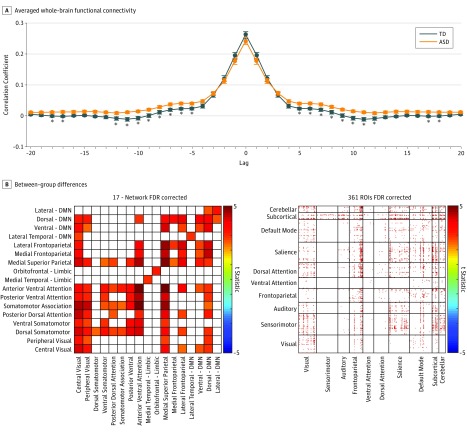
Aberrant Lagged-Based Functional Connectivity in Individuals With Autism A, Comparison of averaged whole-brain functional connectivity between individuals with autism and those in the control group across −20 to +20 lags. Error bars represent standard error values. The asterisks represent between-group differences significant after false discovery rate correction (q[FDR] < .05). B, Distribution of increased functional connectivity in individuals with autism relative to controls at lag 4 (−6.212 seconds) across a 17-network parcellation (q[FDR] < .05) and across 361 gray matter regions of interest (ROIs) (q[FDR] < .05). ASD indicates autism spectrum disorder; DMN, default mode network; and TD, typically developing.

Sustained connectivity analyses revealed significant increases in individuals with autism relative to controls primarily in medial superior parietal, attention, and default mode (DMN) networks. For connections showing significantly higher sustained connectivity, *P* values ranged from less than .01 to .006 (*t*_86_, 2.8-3.6). None of the between-group differences between pairs of the 361 gray matter ROIs reached significance after multiple comparison correction (Figure 1). Averaged functional connectivity values were nonsignificantly increased in the control group compared with autism at 0 lag. From −5 to +5 lags to −12 to +12 lags, averaged functional connectivity values are significantly increased in individuals with autism compared with those in the control group, when controlling for multiple comparisons (*P* values for lags 5-12: .008, .005, .01, .02, .01, .004, .004, and .02; *t* statistic: 2.73, 2.87, 2.61, 2.44, 2.65, 3.0, 2.98, and 2.41) (Figure 2). When comparing between-group functional connectivity at 0 lag, we found significantly decreased synchrony across brain regions in individuals with autism compared with those in the control group primarily in connections between the DMN and limbic networks with somatomotor networks (eFigure 1 in the Supplement). When comparing the same parcellations at lag −4 to +4 (6.212 seconds), we found significantly increased correlation of lagged pairs of time series in individuals with autism compared with those in the control group in widespread gray matter regions when autocorrelation was averaged over all 361 regions (Figure 2). Taken together, these findings suggest that group differences in functional connectivity between brain regions increase as the lag between the time series increases (Video).

**Video.** Prolonged Functional Connections in Autism Lagged functional connectivity in individuals with autism compared with controls across multiple lags.

### Reproducibility of Between-Group Findings

**Figure 3.**
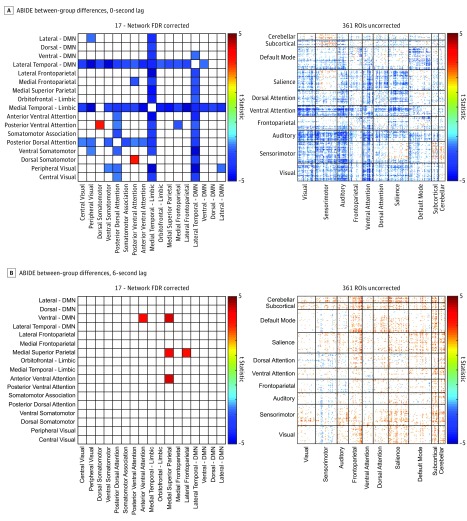
Aberrant Lagged-Based Functional Connectivity in Individuals With Autism in a Replication Sample A, Distribution of decreased functional connectivity in individuals with autism relative to controls at 0 lag across a 17-network parcellation (false discovery rate correction, q[FDR] < .05) and across 361 gray matter regions of interest (ROIs) (uncorrected, *P* < .05). B, Distribution of increased functional connectivity in individuals with autism relative to those in the control group at a 6-second lag across a 17-network parcellation (q[FDR] < .05) and across 361 gray matter ROIs (uncorrected, *P* < .05). ABIDE indicates Autism Brain Imaging Data Exchange; DMN, default mode network.

Significantly decreased functional connectivity was found in individuals with autism compared with those in the control group at 0 lag predominantly between DMN and limbic networks. In the current sample, widespread increased synchrony in individuals with autism emerged at lag −4 to +4 (6.212 seconds) (Figure 3). Therefore, functional connectivity at 6 seconds was also compared between groups in the ABIDE data set. Significantly increased lagged connectivity in autism was found between the medial superior parietal network and ventral DMN and anterior ventral attention networks (Figure 3). Connections that show higher synchrony in autism for lagged time series tend not to show lower synchrony in autism at lag 0, and vice versa, suggesting that while all connections show increases in relative synchrony in autism as lag increases, connections with significant lagged synchrony tend to be those that were not hypoconnected in autism at lag 0. While the direction of the association is similar for ABIDE and high-resolution samples, the specific network connections that were significant differed.

### Behavioral Associations With Sustained Connectivity

In the Utah cohort, individuals with autism were found to have significant widespread correlations between sustained connectivity and SRS-restricted and repetitive behaviors (findings meeting q[FDR] <.05), SRS-social communication and interaction, and SRS total scores (eFigure 2 in the Supplement). All of these relationships remained significant when additionally covarying for peak connectivity, suggesting that these findings are specific for sustained connectivity and not inherited from zero-lag connectivity relationships. No significant relationship between sustained connectivity and ADOS calibrated severity scores or TMT were found in the autism group. In the control group, TMT-B and TMT-B-A were found to be positively associated with sustained connectivity primarily in visual and attentional networks (findings meeting q[FDR] <.05). No significant findings were found for TMT-A (eFigure 3 in the Supplement). A significant increase in TMT completion time was found in individuals with autism compared with those in the control group for the TMT-A, TMT-B, and TMT-B-A. No significant relationships were seen between performance IQ and sustained connectivity for either autism or typically developing samples.

## Discussion

Using extended multiband resting-state fMRI acquisitions, we found increased sustained connectivity in adolescent and adult males with autism that was related to severity in autism symptoms. Increased sustained connectivity in individuals with autism was evident across the cortex, subcortical gray matter, and cerebellar networks, with significant between-group differences between time series from pairs of 17 intrinsic connectivity networks. Whereas our sample showed variable increases and decreases across the brain in functional connectivity at 0 lag, consistent with findings from the literature, there was a shift toward consistently increased connectivity for lagged time series between regions that was seen across the brain both in our sample with high-temporal resolution imaging data as well as in the ABIDE sample.

The TMT completion time, a metric associated with slower processing speed, was significantly increased in individuals with autism compared with those in the control group in our sample. Increased processing speed in autism were detailed in the literature^18^; however, divergent findings have also been reported.^42^ In the control group, sustained connectivity was positively associated with TMT-B, a measure of cognitive flexibility, and primarily TMT-B-A. One interpretation of increased TMT-B-A completion times suggests decreased processing speed.^43^ These findings are in line with a previous report that found a negative correlation between processing speed and sustained connectivity.^16^ Taken together, these data suggest that individuals with limited cognitive flexibility and slower processing speeds may exhibit functional cortical connections that last longer than those with faster processing speed.

No significant correlations were found between measures of cognitive function and sustained connectivity in individuals with autism. However, a significant positive correlation was found between sustained connectivity and social impairment scores. Similarly, Rashid et al^14^ found a link between dynamic functional connectivity and autistic traits assessed using the SRS. These findings also dovetail with electrophysiological results showing delayed auditory evoked responses in autism suggesting temporal prolongation of steady-state responses to stimuli.^44^ Temporal smoothing of neural responses with prolonged brain states may also be consistent with another hypothesized physiological mechanism in autism, that of decreased ability for shifting of attention.^45^

Previous studies of dynamic functional connectivity in autism have pointed to critical differences in the temporal components of connectivity in autism. In a study using a sliding-window approach to dynamic connectivity, increased intraindividual variability over time was observed, indicating less rapid switching between networks or states at somewhat longer temporal scales than we measured.^11,14^ A recent report investigating lag structure in autism demonstrated differences in the relative lag of individual regions across the brain compared with typically developing control participants, additionally suggesting specific differences in lagged connectivity.^46^

Sustained or lagged connectivity using fMRI data are influenced by the synchrony of neural responses combined with other factors influencing the hemodynamic response of the BOLD signal such as blood flow^47^ and BOLD signal transduction.^48^ A potential contribution of vascular physiology in the between-group differences we observe is not excluded, although a previous study compared sustained connectivity with the width of the regional hemodynamic response function, demonstrating a close correlation with the hemodynamic response function that itself was associated with individual differences in cognition including processing speed.^16^ Observed individual variation in hemodynamic response^49,50^ may actually represent effects of sustained neural oscillations or activity that differ between individuals and spatial locations rather than differences in vascular components. A testable hypothesis from our results would be that the width of correlation curves (including signal autocorrelation) of band-limited power in electrophysiological signals such as magnetoencephalography or electroencephalography would also be increased in autism.

Prolonged synchrony of brain connections in autism may constrain potential neurobiological hypotheses of pathophysiological mechanisms. In particular, the hypothesis that autism may be related to an imbalance in excitation vs inhibition in local circuitry^51,52,53^ might be consistent with persistent oscillations in local circuitry associated with decreased inhibitory drive. This could be further evaluated by electrophysiological demonstration of persistent lagged synchrony or evaluation of the genetic basis of sustained connectivity, which has been shown to be heritable,^16^ in relation to synaptic mechanisms underlying excitatory vs inhibitory balance.

### Limitations

The neurophysiological mechanism by which sustained connectivity is prolonged in autism will require further research, and the relative contribution of vascular differences compared with differences in neural circuitry remains unclear. Alternately, prolonged connectivity may reflect changes in the frequency distribution of the BOLD signal in autism, with shift to lower frequencies. Sustained connectivity, like traditional functional connectivity, is variable across participants and may have limited single-participant diagnostic or prognostic value given the extreme heterogeneity of autism. Effects of medication use may also contribute to observed differences. Developmental changes in sustained connectivity with age may be incompletely modeled even after covarying the results with age in our analyses, and future research will be needed to determine the extent to which differential sustained connectivity in autism is age dependent.

## Conclusions

Sustained connectivity and other temporal metrics probing the duration of functional synchrony between brain regions provide pathophysiological hypotheses of autism. Further research into the temporal dynamics of resting-state functional connectivity may reconcile heterogeneous and inconsistent findings of functional connectivity in autism and could be evaluated in other neurodevelopmental and neuropsychiatric disorders.

## Notes

Supplement. **eTable.** List of Research Sites Included in the ABIDE Dataset **eFigure 1.** Aberrant Functional Connectivity in Individuals With Autism **eFigure 2.** Sustained Connectivity is Positively Associated With Autistic Traits **eFigure 3.** Sustained Connectivity is Negatively Associated With Trail Making Completion Time
