# Supplementary material for: Evaluation of Differences in Temporal Synchrony Between Brain Regions in Individuals With Autism and Typical Development
Source: JAMA Netw Open. 2018 Nov 16;1(7):e184777. doi: 10.1001/jamanetworkopen.2018.4777 (PMC6324391; doi:10.1001/jamanetworkopen.2018.4777)
Supplement: Supplement. — eTable. List of Research Sites Included in the ABIDE Dataset eFigure 1. Aberrant Functional Connectivity in Individuals With Autism eFigure 2. Sustained Connectivity is Positively Associated With Autistic Traits eFigure 3. Sustained Connectivity is Negatively Associated With Trail Making Completion Time [file jamanetwopen-1-e184777-s001.pdf]

Supplementary Online Content

King JB, Prigge MBD, King CK, et al. Evaluation of differences in temporal synchrony between brain regions in individuals with autism and typical development. *JAMA Netw Open*. 2018;1(7):e184777. doi:10.1001/jamanetworkopen.2018.4777

eTable. List of Research Sites Included in the ABIDE Dataset
eFigure 1. Aberrant Functional Connectivity in Individuals With Autism
eFigure 2. Sustained Connectivity is Positively Associated With Autistic Traits
eFigure 3. Sustained Connectivity is Negatively Associated With Trail Making Completion Time

This supplementary material has been provided by the authors to give readers additional information about their work.

© 2018 JB King et al. *JAMA Network Open*.

eTable. List of Research Sites Included in the ABIDE Dataset

**ABIDE I Sites**	
KKI	Kennedy Krieger Institute
Leuven	University of Leuven
MaxMun	Ludwig Maximilian University of Munich
NYU	New York University Langone Medical Center
Pitt	University of Pittsburgh School of Medicine
SDSU	San Diego State University
Trinity	Trinity Centre for Health Sciences
UCLA	University of California Los Angeles
UM	University of Michigan
USM	University of Utah School of Medicine
Yale	Yale Child Study Center
**ABIDE II Sites**	
EMC	Eramus University Medical Center
GU	Georgetown University
IP	Institut Pasteur and Robert Debré Hospital
IU	Indiana University
KKI	Kennedy Krieger Institute
NYU	New York University Langone Medical Center
OHSU	Oregon Health and Science University
ONRC	Olin Neuropsychiatry Research Center, Institute of Living at Hartford Hospital
SDSU	San Diego State University
Stanford	Stanford University
TCD	Trinity Centre for Health Sciences
UCD	University of California Davis
UCLA	University of California Los Angeles
USM	University of Utah School of Medicine

© 2018 JB King et al. *JAMA Network Open*.

eFigure 1. Aberrant Functional Connectivity in Individuals With Autism

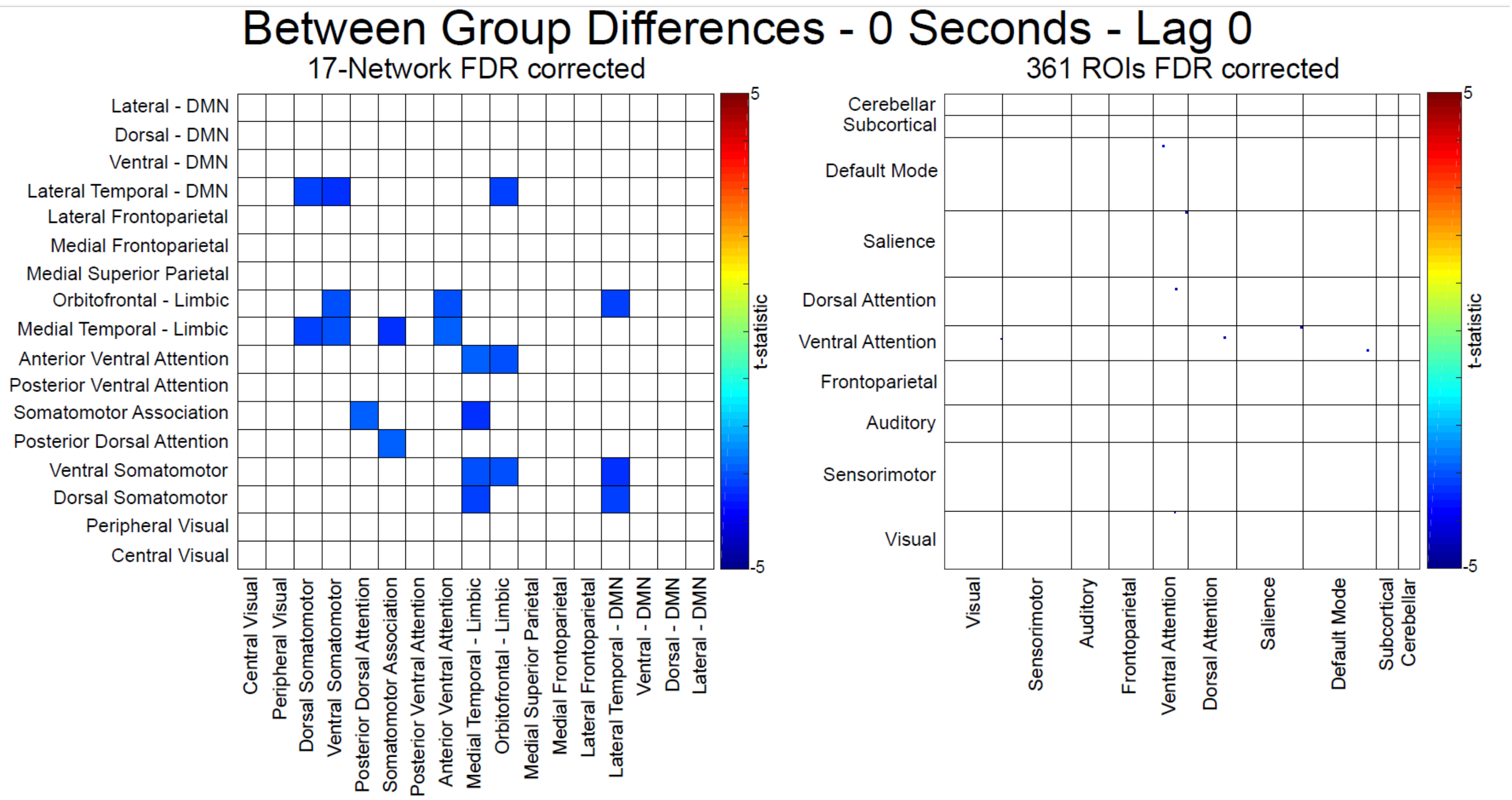

Distribution of decreased functional connectivity in individuals with autism relative to controls at zero lag across a 17-network parcellation (false disovery rate correction, q[FDR] < .05) and across 361 gray matter regions of interest (ROI) (q[FDR] < .05).

© 2018 JB King et al. *JAMA Network Open*.

eFigure 2. Sustained Connectivity is Positively Associated With Autistic Traits

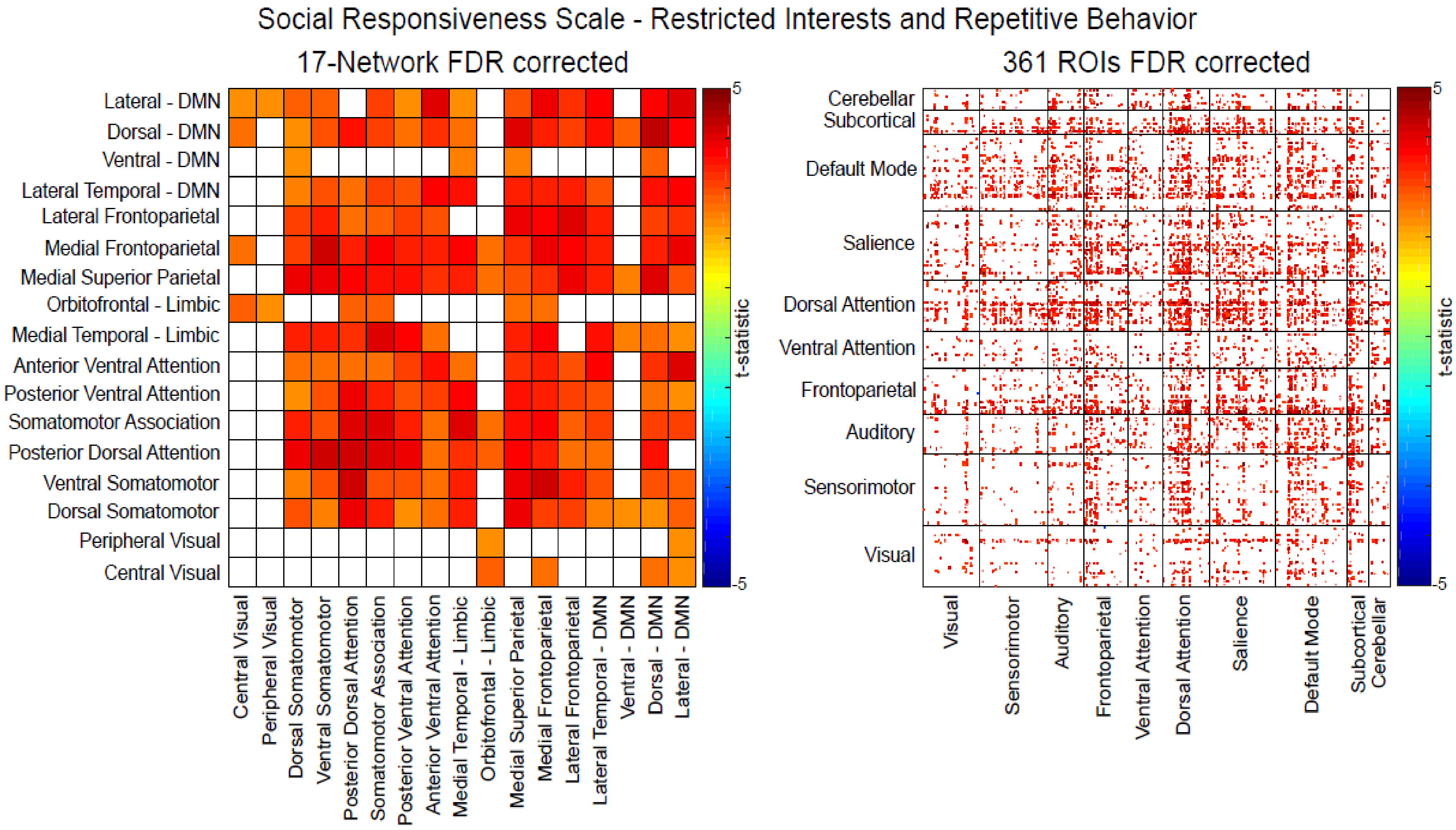

© 2018 JB King et al. *JAMA Network Open*.

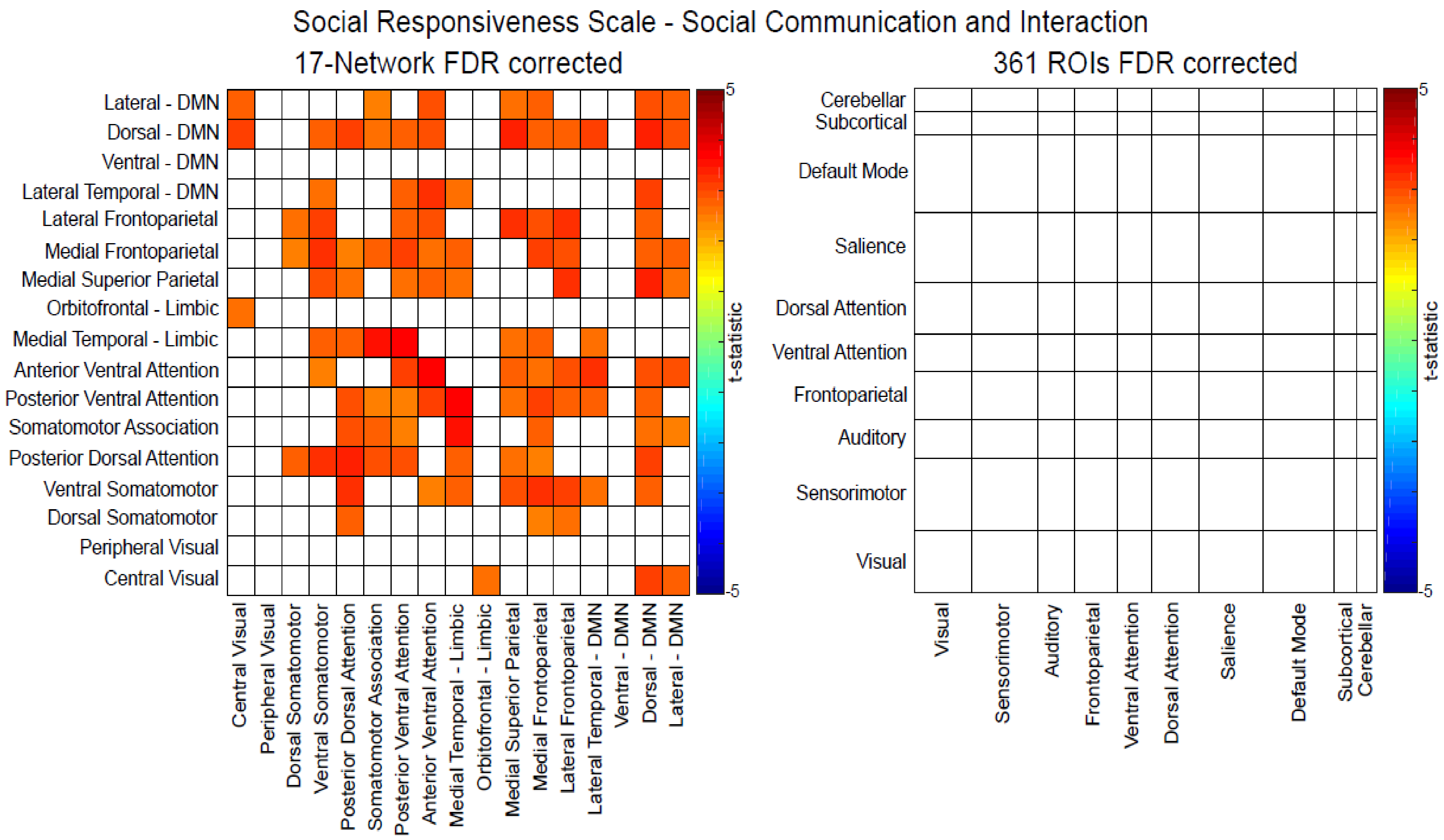

© 2018 JB King et al. *JAMA Network Open*.

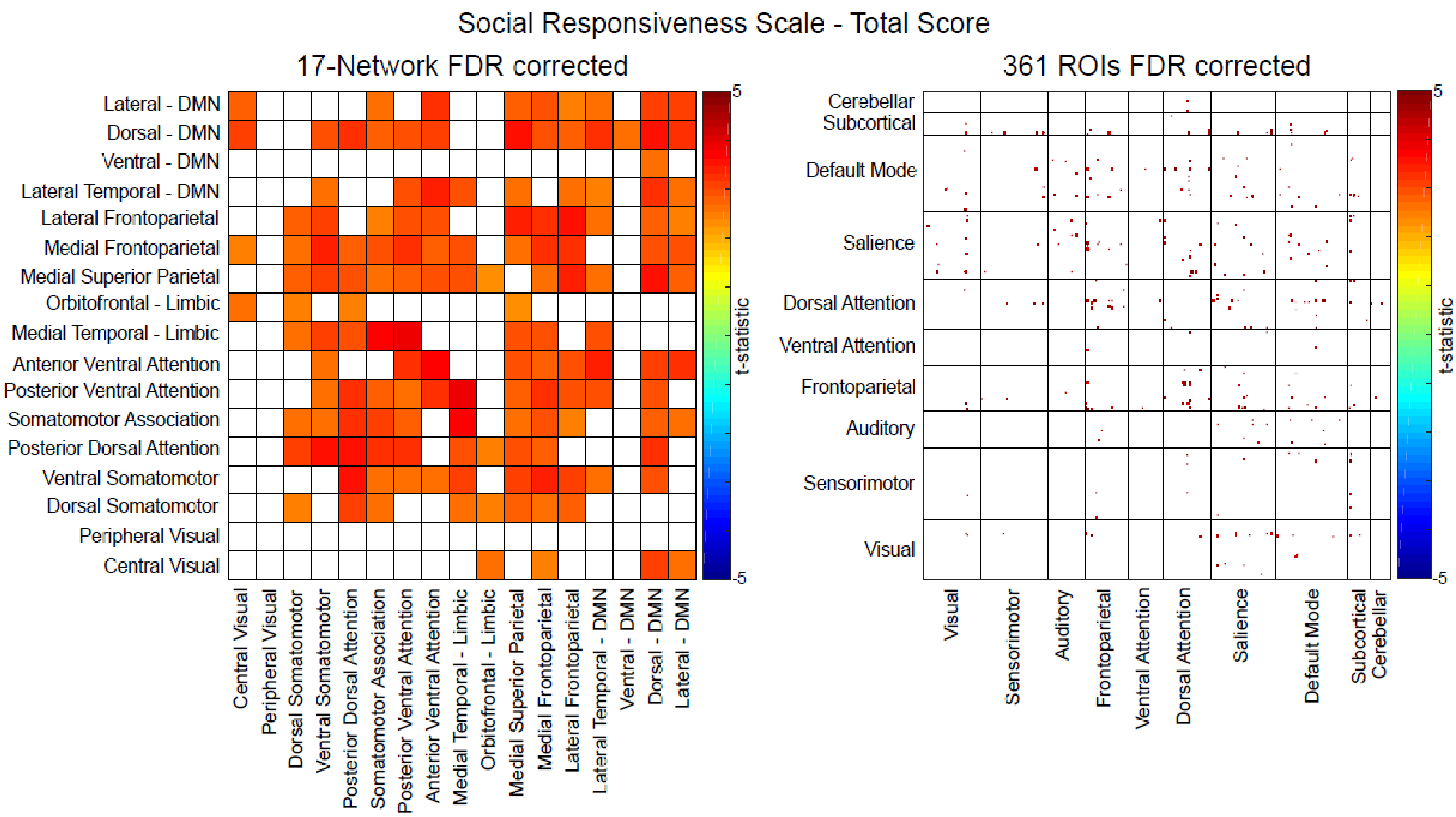

A significant (q[FDR] < .05) relationship was found between Social Responsiveness Scale scores and autistic traits in individuals with autism across a 17-network parcellation and across 361 gray matter regions of interest (ROI). DMN = Default Mode Network.

© 2018 JB King et al. *JAMA Network Open*.

eFigure 3. Sustained Connectivity is Negatively Associated With Trail Making Completion Time

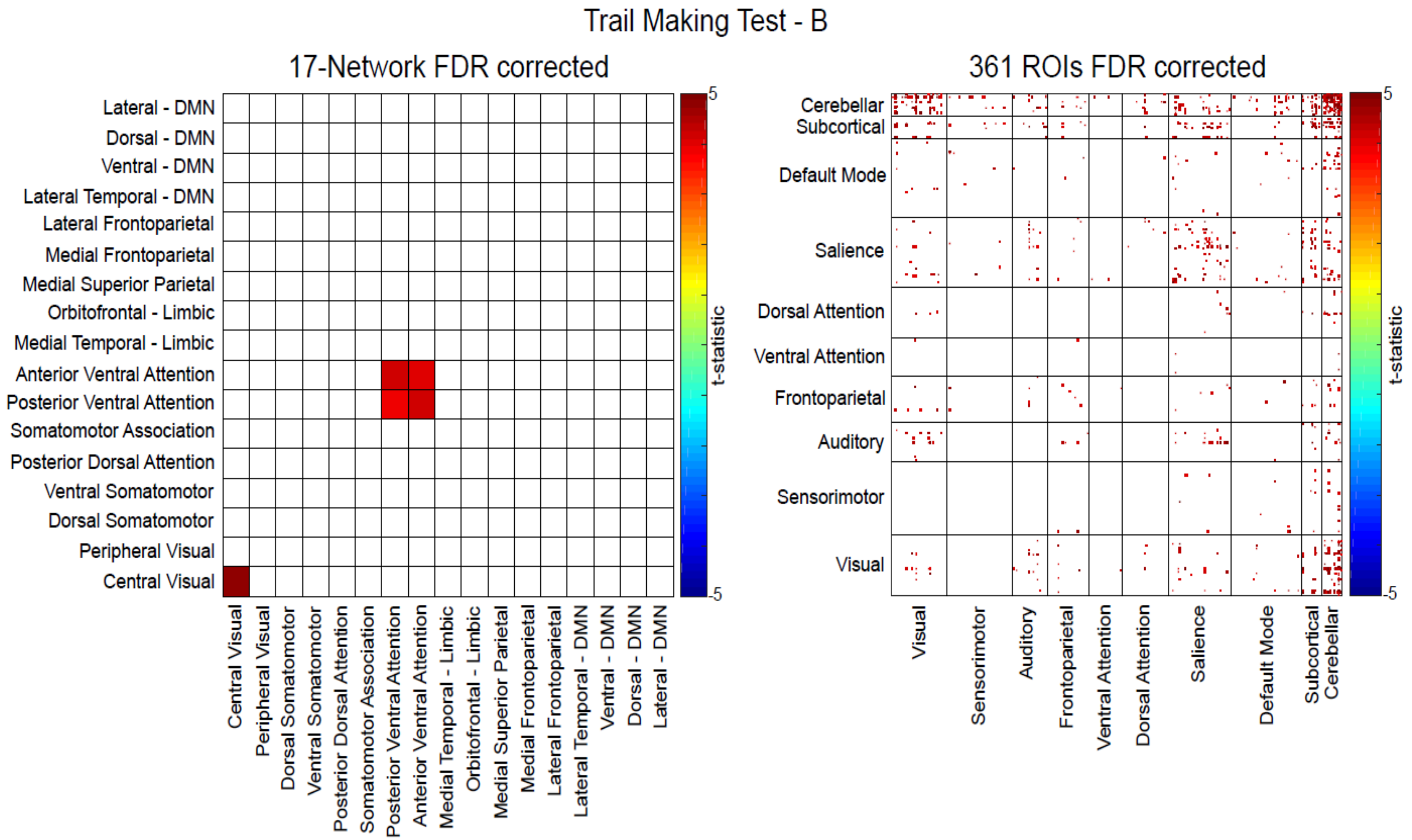

© 2018 JB King et al. *JAMA Network Open*.

–

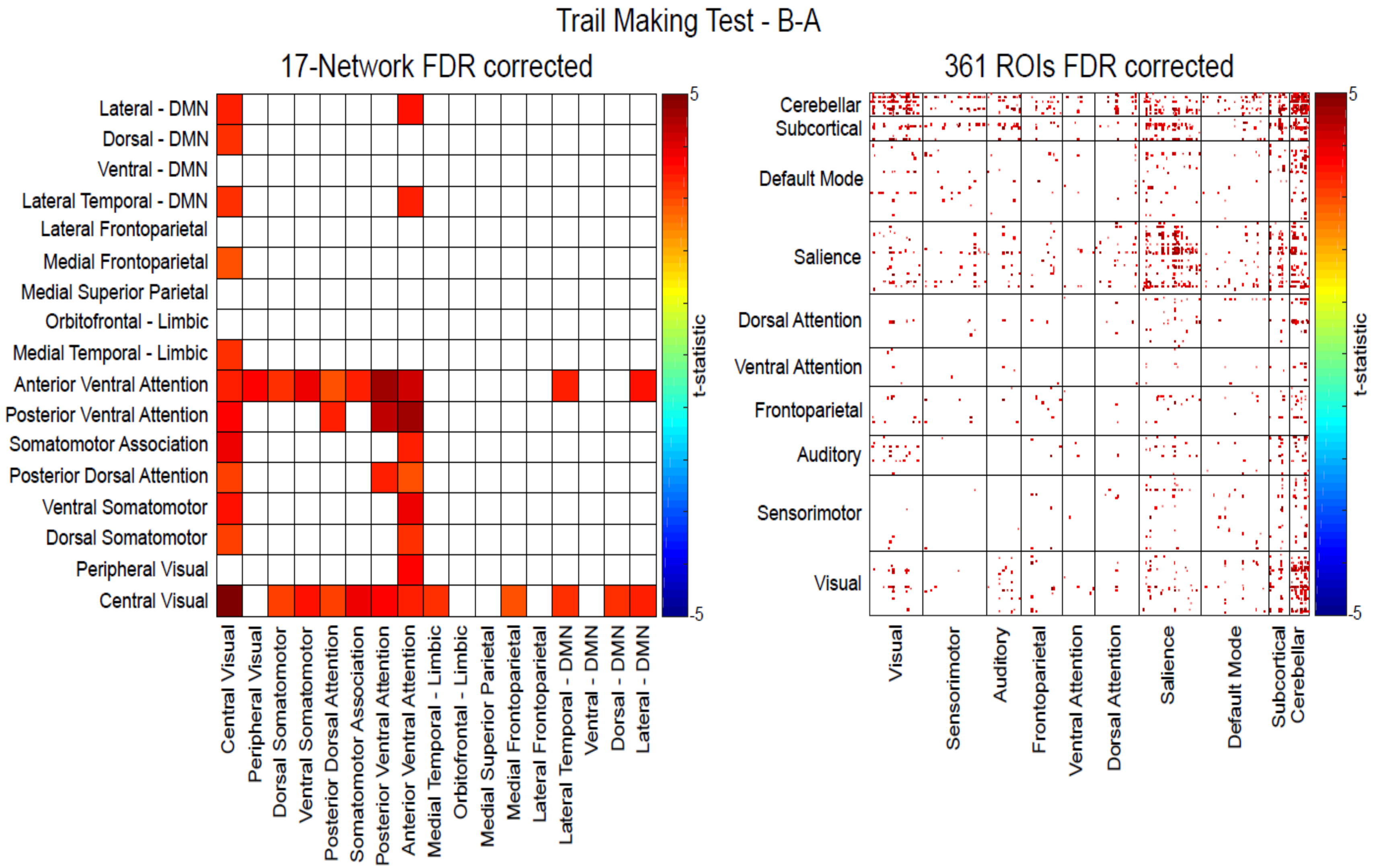

© 2018 JB King et al. *JAMA Network Open*.

In typically developing individuals, Trail Making Test Part B and Part B minus A completion times, a measure of processing speed where higher times indicate poorer processing speeds, were positively associated with sustained connectivity across a17-network parcellation (q[FDR] < .05) and across 361 gray matter regions of interest (ROI) with false discovery rate q[FDR] < .05 suggesting an inverse relationship between processing speed and sustained connectivity. DMN = Default Mode Network.

© 2018 JB King et al. *JAMA Network Open*.
